# Improving quality of care and clinical outcomes for rectal cancer through clinical audits in a multicentre cancer care organisation

**DOI:** 10.1186/s13014-020-1465-z

**Published:** 2020-01-31

**Authors:** M. G. Torras, E. Canals, C. Muñoz-Montplet, A. Vidal, D. Jurado, A. Eraso, S. Villà, M. Caro, J. Molero, M. Macià, M. Puigdemont, E. González-Muñoz, A. López, F. Guedea, J. M. Borras

**Affiliations:** 1 Clinical Management Department, Institut Català d’Oncologia, Barcelona, Spain; 2 Radiation Oncology Department, Institut Català d’Oncologia, Girona, Spain; 3 Medical Physics and Radiation Protection Department, Institut Català d’Oncologia, Girona, Spain; 4 Quality and Results Department, Institut Català d’Oncologia, Girona, Spain; 5 Medical Physics and Radiation Protection Department, Institut Català d’Oncologia, Girona, Spain; 6 Radiation Oncology Department, Institut Català d’Oncologia, Badalona, Spain; 7 Medical Physics and Radiation Protection Department, Institut Català d’Oncologia, Girona, Spain; 8 Radiation Oncology Department, Institut Català d’Oncologia, Hospitalet del Llobregat, Barcelona, Spain; 9 Hospital Tumor Registry, Institut Català d’Oncologia, Girona, Spain; 10 Cancer Prevention and Control Program, Institut Català d’Oncologia, Hospitalet del Llobregat, Barcelona, Spain; 11 Radiation Oncology Department, Institut Català d’Oncologia, Barcelona, Spain; 12 Department of Clinical Sciences, IDIBELL, University of Barcelona, Barcelona, Spain

**Keywords:** Rectal Cancer, Clinical audit, Radiotherapy process, Clinical practice variability, Improvement Quality process

## Abstract

**Introduction:**

Colorectal cancer treatment requires a complex, multidisciplinary approach. Because of the potential variability, monitoring through clinical audits is advisable. This study assesses the effects of a quality improvement action plan in patients with locally advanced rectal cancer and treated with radiotherapy.

**Methods:**

Comparative, multicentre study in two cohorts of 120 patients each, selected randomly from patients diagnosed with rectal cancer who had initiated radiotherapy with a curative intent. Based on the results from a baseline clinical audit in 2013, a quality improvement action plan was designed and implemented; a second audit in 2017 evaluated its impact.

**Results:**

Standardised information was present on 77.5% of the magnetic resonance imaging (MRI) staging reports. Treatment strategies were similar in all three study centres. Of the patients whose treatment was interrupted, just 9.7% received a compensation dose. There was an increase in MRI re-staging from 32.5 to 61.5%, and a significant decrease in unreported circumferential resection margins following neoadjuvant therapy (ypCRM), from 34.5 to 5.6% (*p* <  0.001).

**Conclusions:**

The comparison between two clinical audits showed improvements in neoadjuvant radiotherapy in rectal cancer patients. Some indicators reveal areas in need of additional efforts, for example to reduce the overall treatment time.

## Introduction

Colorectal cancer is the third most common cancer worldwide and the second in Catalonia; 45% of these tumours are in the rectum [1, 2]. A locally advanced rectal tumour (LART) is defined by the presence of a neoplasm classified as T3/T4, with or without lymph node infiltration (N0/N+), and no metastatic dissemination. Treatment is multidisciplinary and consists of surgery plus radiochemotherapy [3], with careful evaluation of magnetic resonance imaging (MRI) parameters before and after preoperative treatment. The total mesorectal excision is a quality indicator, and the main factor for local control, but in LART the neoadjuvant treatment achieves greater control than surgery alone. Since the results of the Swedish Rectal Cancer Trial were published in 1997 [4], neoadjuvant, multimodal treatment has taken root as the standard treatment strategy. Substantial reductions in local recurrence have been achieved thanks to radiotherapy (RT), both short-course radiotherapy (SCRT) and long-course radiotherapy (LCRT), which is combined with chemotherapy. In historical series, local recurrence was 30 to 45%, compared with less than 10% today [5]. In 2019, multimodality treatment for LART also implies personalised strategies to decrease the surgical burden for patients responding after pre-operative treatment, including ‘wait and watch’ or organ-preservation approaches.

Thus, successful treatment of locally advanced rectal cancer requires a multidisciplinary approach [6, 7]. The complexity of care processes – and the resulting potential for variability in clinical decision-making – make monitoring highly desirable. Clinical audits, for their part, are a widely used instrument to identify variability in clinical practice and areas in need of improvement. Several countries have incorporated audits as a strategy to evaluate health policies [8, 9]. The impact of the results obtained depends to a large extent on the analysis of the outcomes, the quality of the data collected, and the credibility of the audit team. There are few studies that report the effects of corrective measures established based on post-intervention audits [10].

The aim of this study was to assess the effects of the quality improvement action plan on the quality of clinical care processes in patients with rectal cancer, with an emphasis on the diagnostic and neoadjuvant treatment phases.

## Methods

This study took place in the Institut Català d’Oncologia (ICO), a cancer care organisation with three specialised centres offering radiation oncology treatments. ICO collaborates with the corresponding university hospitals that belong to the Institut Calatà de la Salut (ICS);it works in a network with another 17 more local hospitals, and it is the reference centre in oncology for nearly 3 million people (45% of the adult population in Catalonia). The care model is characterised by a multidisciplinary focus on specific pathologies, in compliance with corporate clinical practice guidelines.

### Study design

This multicentre comparative study was performed over two periods (2013 and 2017). For each study year, we selected a representative sample of patients diagnosed with rectal cancer and treated with neoadjuvant RT.

The study began with the creation of a working group made up of radiation oncologists and medical physicists from all three centres, and led by the clinical reference centre in rectal cancer. Key aspects of the diagnostic, therapeutic and follow-up phases were identified based on the literature [11, 12], as were the study indicators, including the appropriateness of the diagnostic tests performed, quality of the diagnostic reports, multidisciplinary approach, appropriateness and adherence to the prescribed treatment, imaging controls during the RT sessions, post-surgical circumferential resection margin (ypCRM), and adverse effects. A purpose-designed form was used to collect the required data during the first audit, and for the second audit it was adapted to include the new indicators derived from the action plan.

The first clinical audit for radiation oncology (ACOR-1) identified areas in need of improvement. The analysis of these published results [13] informed the development of an action plan, which was approved in April 2016 and implemented thereafter. A new audit (ACOR-2) was then performed to assess the extent to which the improvement targets were achieved.

The audits consisted of a review of a random sample of 40 clinical histories from each centre. The target population was patients diagnosed with rectal cancer (International Classification of Diseases; ICD-9:154.1) who received neoadjuvant RT with a curative intent (starting in 2013 in the first cohort and between June and December of 2017 in the second).

For ACOR-2, the cognizant clinical practice guidelines specified the criteria for concomitant chemotherapy and two alternative strategies for RT: LCRT (45 Gy to 50.4 Gy in fractions of 1.8 Gy) and SCRT (25 Gy in fractions of 5 Gy), administered on 5 consecutive days. The SCRT strategy was preferentially indicated in patients over the age of 70, in tumours of the middle third of the rectum, and in disease without involvement of the mesorectal fascia or metastasis [14].

Following a pilot test, a team of external auditors undertook the first audit from June to September 2015, and the second from June to September 2018. Data were collected from the electronic clinical records in the participating centres (SAP i.s.h. med Cerner & SAP) and the specific information system for radiotherapy (Aria oncology information System BY Varian Medical Systems Inc.).

### Action plan

For the diagnostic phase, the quality improvement action plan (Table 1**)** covered measures to increase the available clinical information needed to develop a treatment strategy, based on personal communication and multidisciplinary committees. The audit highlighted the need to standardise the MRI report, based on the Mercury Study Group criteria [15]. Another aspect considered was tumour board review to approve the treatment plan, in patients diagnosed both in our centres and in the regional hospitals.

**Table 1:** Description of action plan (approved 18 April 2016)

Area for improvement	Target
Diagnostic phase	
1 Increase availability of biopsy report	> 95% availability of reports
2 Standardise MRI report	> 85% of reports meet Mercury Study Group criteria* (ICS-IDI diagnostic imaging service)
3 Increase multidisciplinary working (treatment strategy)	> 85% of patients diagnosed at ICO have cases reviewed by tumour board
Treatment phase	
1 Standardise criteria for indicating radiotherapy (IMRT)†	> 85% of patients with indication for radiotherapy are treated with IMRT
2 Improve adherence to planned RT treatment (OTT)	
Develop protocol for managing treatment interruptions	Approve protocol by October 2016 and implement it
Disseminate and apply protocol	Reduce % of patients with prolonged OTT
	Reduce % of patients finalising treatment on Monday
3 Standardise criteria for imaging controls during treatment	
Develop protocol for imaging controls during treatment (IGRT)	Approve protocol by October 2016 and implement it
Disseminate and apply protocol	> 85% of patients with imaging control according to protocol
4 Standardise anatomical pathology report (surgical specimen): report ypCRM	> 85% of pathology reports note ypCRM
Follow-up phase	
1 Report adverse effects according to severity, based on CTCAE (v4.0) classifications	> 85% of follow-up visits record presence or absence of adverse events and their CTCAE grade during the clinical course

*CTCAE* Common Terminology Criteria for Adverse Events, *ICS* Institut Català de la Salut, *ICO* Institut Català d’Oncologia, *IDI* Institut de Diagnòstic per la Imatge, *IGRT* image-guided radiotherapy, *IMRT* intensity-modulated radiotherapy, *MRI* magnetic resonance imaging, *OTT* overall treatment time, *ypCRM* circumferential resection margin (following neoadjuvant treatment)

^a^Mercury Study Group criteria:

1) distance to anal verge and relationship between tumour and sphincter

2) infiltration beyond muscularis propia in mm

3) lymphatic state

4) extramural vascular spread

5) potential CRM invasion

6) presence of tumour implants

^b^Indication for IMRT according to patient profile:

1) tumour in lower third of rectum in which dosage to genitals should be reduced to V30 < 35%

2) rectal tumour in patients with hysterectomy (or laparotomy) in whom the dosage to the intestine exceeds V15 < 150 cc or V15 < 120 cc

3) rT4 rectal tumour with infiltration to prostate, urinary bladder or uterus, in whom the planning target volume includes lymphatic areas of the external iliac system

Measures to improve the treatment phase included standardising the use of intensity-modulated radiotherapy (IMRT) in the three centres, based on the following indications: tumours in the lower third of the rectum; patients with hysterectomy; rT4 rectal tumours; and infiltration to the prostate, bladder or uterus in patients in whom the planning target volume included the lymphatic areas of the external iliac system lymph nodes [16]. Other measures were the development and implementation of a management protocol for RT session interruptions to reduce prolonged overall treatment time (OTT) and a protocol for image-guided radiotherapy (IGRT) during the treatment (to be undertaken at least once a week); an increase in the percentage of patients re-evaluated with MRI prior to surgery; and the state of the ypCRM on the pathology report describing the surgical specimens.

Finally, for the follow-up, the action plan aimed to improve reporting of adverse effects over the clinical course, according to the Common Terminology Criteria for Adverse Events (CTCAE) v4.0 [17].

We used R software available as Free Software (version 3.0.2) for the statistical analysis. The sample size calculation was based on a level of confidence of 0.95 (α = 0.05), assuming a finite population of about 100 cases per centre and a 20% rate of attrition. Pearson’s chi-squared test was used to compare results between groups.

## Results

Table 2 presents the characteristics of the patients included in the ACOR-1 and ACOR-2 cohorts. There were no significant differences in the demographic or clinical profiles, except in histological grade and distance from tumour to mesorectal fascia. Table 3 shows the outcomes for the diagnostic phase after implementing the action plan. Availability of the biopsy report reached 100% in patients in the second period, as did MRI staging in two of the three centres. The MRI reports met the established criteria in 77.5% of the cases, representing a significant rise with respect to the first period. In centre A, there were 10 cases where the report noted the distance from the tumour to the anorectal junction rather than the anal verge (these measures are considered equivalent with respect to the fulfilment of the standard).

**Table 2:** Patient’s clinical characteristics

	Total patients			Centre A			Centre B			Centre C		
	*ACOR-1* *(N = 120)*	*ACOR-2* *(N = 120)*	*p-value*	*ACOR-1* *(N = 40)*	*ACOR-2* *(N = 40)*	*p-value*	*ACOR-1* *(N =* 40*)*	*ACOR-2* *(N = 40)*	*p-value*	*ACOR-1* *(N = 40)*	*ACOR-2* *(N = 40)*	*p-value*
Sex												
Men	86 (71.7%)	85 (70.8%)	*> 0.99*	29 (72.5%)	25 (62.5%)	0.47	27 (67.5%)	32 (80%)	0.31	30 (75%)	28 (70%)	0.80
Women	34 (28.3%)	35 (29.2%)		11 (27.5%)	15 (37.5%)		13 (32.5%)	8 (20%)		10 (25%)	12 (30%)	
Age (years)												
< 60	28 (23.3%)	42 (35%)	0.13	13 (32.5%)	12 (30%)	0.59	9 (22.5%)	15 (37.5%)	0.45	6 (15%)	15 (37.5%)	0.15
60–69	42 (35%)	37 (30.8%)		11 (27.5%)	13 (32.5%)		16 (40%)	12 (30%)		15 (37.5%)	12 (30%)	
70–79	40 (33.3%)	28 (23.3%)		13 (32.5%)	9 (22.5%)		13 (32.5%)	10 (25%)		14 (35%)	9 (22.5%)	
≥ 80	10 (8.3%)	13 (10.8%)		3 (7.5%)	6 (15%)		2 (5%)	3 (7.5%)		5 (12.5%)	4 (10%)	
Histology												
Invasive adenocarcinoma	119 (99.2%)	120 (100%)	*> 0.99*	40 (100%)	40 (100%)	*> 0.99*	40 (100%)	40 (100%)	*> 0.99*	39 (97.5%)	40 (100%)	*> 0.99*
Squamous cell carcinoma	1 (0.8%)	0 (0%)		0 (0%)	0 (0%)		0 (0%)	0 (0%)		1 (2.5%)	0 (0%)	
Histological grade												
Well differentiated	43 (35.8%)	51 (42.5%)	0.37	9 (22.5%)	3 (7.5%)	0.01†	17 (42.5%)	18 (45%)	0.60	17 (42.5%)	30 (75%)	0.015†
Moderately differentiated	20 (16.7%)	25 (20.8%)		3 (7.5%)	14 (35%)		7 (17.5%)	9 (22.5%)		10 (25%)	2 (5%)	
Poorly differentiated	4 (3.3%)	2 (1.7%)		1 (2.5%)	0 (0%)		0 (0%)	1 (2.5%)		3 (7.5%)	1 (2.5%)	
NR	53 (44.2%)	42 (35%)		27 (67.5%)	23 (57.5%)		16 (40%)	12 (30%)		10 (25%)	7 (17.5%)	
Distance from tumour to mesorectal fascia												
≤ 1 mm	29 (24.2%)	43 (35.8%)	0.001‡	9 (22.5%)	13 (32.5%)	0.24	14 (35%)	14 (35%)	0.07	6 (15%)	16 (40%)	0.01†
> 1 mm	45 (37.5%)	56 (46.7%)		14 (35%)	17 (42.5%)		19 (47.5%)	25 (62.5%)		12 (30%)	14 (35%)	
Not reported	46 (38.3%)	21 (17.5%)		17 (42.5%)	10 (25%)		7 (17.5%)	1 (2.5%)		22 (55%)	10 (25%)	
Distance from tumour to anal verge												
0–5 cm	24 (29.3%)	37 (35.9%)	0.51	2 (22.2%)	8 (32%)	0.15	13 (33.3%)	13 (33.3%)	*> 0.99*	9 (26.5%)	16 (41%)	0.42
> 5–10 cm	41 (50%)	43 (41.7%)		7 (77.8%)	11 (44%)		15 (38.5%)	15 (38.5%)		19 (55.9%)	17 (43.6%)	
> 10 cm	17 (20.7%)	23 (22.3%)		0 (0%)	6 (24%)		11 (28.2%)	11 (28.2%)		6 (17.6%)	6 (15.4%)	
Stage												
I	2 (1.7%)	0 (0%)	0.38	0 (0%)	0 (0%)	0.95	1 (2.5%)	0 (0%)	0.384	1 (2.5%)	0 (0%)	0.679
II	14 (11.7%)	10 (8.3%)		7 (17.5%)	6 (15%)		4 (10%)	1 (2.5%)		3 (7.5%)	3 (7.5%)	
III	94 (78.3%)	97 (80.8%)		30 (75%)	31 (77.5%)		31 (77.5%)	34 (85%)		33 (82.5%)	32 (80%)	
IV	10 (8.3%)	13 (10.8%)		3 (7.5%)	3 (7.5%)		4 (10%)	5 (12.5%)		3 (7.5%)	5 (12.5%)	

*ACOR* clinical audit for radiation oncology, *ICS* Institut Català de la Salut, *ICO* Institut Català d’Oncologia, *NR* not reported

*p-value 0.05 to 0.01; †*p*-value ≤0.01; ‡*p*-value ≤0.001

**Table 3:** Action plan results: outcomes in diagnostic phase

	Total patients			Centre A			Centre B			Centre C		
	*ACOR-1* *(N = 120)*	*ACOR-2* *(N = 120)*	*p-value*	*ACOR-1* *(N = 40)*	*ACOR-2* *(N = 40)*	*p-value*	*ACOR-1* *(N = 40)*	*ACOR-2* *(N = 40)*	*p-value*	*ACOR-1* *(N = 40)*	*ACOR-2* *(N = 40)*	*p-value*
Was the patient diagnosed outside of ICO-ICS?												
Yes	71 (59.2%)	100 (83.3%)	< 0.001‡	10 (25%)	29 (72.5%)	< 0.001‡	30 (75%)	37 (92.5%)	0.069*	31 (77.5%)	34 (85%)	0.57
No	49 (40.8%)	20 (16.7%)		30 (75%)	11 (27.5%)		10 (25%)	3 (7.5%)		9 (22.5%)	6 (15%)	
In patients diagnosed in ICO-ICS, did a tumour board review the case?												
Yes	39 (88.6%)	20 (100%)	0.29	25 (86.2%)	11 (100%)	0.48	10 (100%)	3 (100%)	0.052	4 (80%)	6 (100%)	0.92
No	5 (11.4%)	0 (0%)		4 (13.8%)	0 (0%)		0 (0%)	0 (0%)		1 (20%)	0 (0%)	
In patients diagnosed outside of ICO-ICS, did a tumour board review the case?												
Yes	34 (47.9%)	68 (68%)	0.019*	3 (30%)	17 (58.6%)	0.23	18 (60%)	35 (94.6%)	0.002	13 (41.9%)	16 (47.1%)	0.55
No	36 (50.7%)	32 (32%)		7 (70%)	12 (41.4%)		12 (40%)	2 (5.4%)		17 (54.8%)	18 (52.9%)	
NR	1 (1.4%)	0 (0%)		0 (0%)	0 (0%)		0 (0%)	0 (0%)		1 (3.2%)	0 (0%)	
Was the biopsy report in the medical history?												
Yes	110 (91.7%)	120 (100%)	0.004†	37 (92.5%)	40 (100%)	0.24	37 (92.5%)	40 (100%)	0.24	36 (90%)	40 (100%)	0.12
No	10 (8.3%)	0 (0%)		3 (7.5%)	0 (0%)		3 (7.5%)	0 (0%)		4 (10%)	0 (0%)	
Was the pelvic MRI recorded in the diagnostic report?												
Yes	114 (95%)	119 (99.2%)	0.13	38 (95%)	40 (100%)	0.47	40 (100%)	40 (100%)	> 0.99	36 (90%)	39 (97.5%)	0.36
No	6 (5%)	1 (0.8%)		2 (5%)	0 (0%)		0 (0%)	0 (0%)		4 (10%)	1 (2.5%)	
Did the MRI report include information on the distance to the anal verge or to the anorectal union**?												
Yes	82 (68.3%)	116 (96.7%)	< 0.001^‡^	9 (22.5%)	38 (95%)	0.001‡	39 (97.5%)	39 (97.5%)	> 0.99	34 (85%)	39 (97.5%)	0.12
No	38 (31.7%)	4 (3.3%)		31 (77.5%)	2 (5%)		1 (2.5%)	1 (2.5%)		6 (15%)	1 (2.5%)	
Did the MRI report include information on the distance to the mesorectal fascia?												
Yes	74 (61.7%)	99 (82.5%)	< 0.001^‡^	23 (57.5%)	30 (75%)	0.16	33 (82.5%)	39 (97.5%)	0.062	18 (45%)	30 (75%)	0.012*
No	46 (38.3%)	21 (17.5%)		17 (42.5%)	10 (25%)		7 (17.5%)	1 (2.5%)		22 (55%)	10 (25%)	
Did the MRI report include enough information to assign a ‘T’ value?												
Yes	112 (93.3%)	117 (98.3%)	0.11	37 (92.5%)	39 (97.5%)	0.61	39 (97.5%)	40 (100%)	> 0.99	36 (90%)	38 (97.4%)	0.37
No	8 (6.7%)	2 (1.7%)		3 (7.5%)	1 (2.5%)		1 (2.5%)	0 (0%)		4 (10%)	1 (2.6%)	
Did the MRI report include enough information to assign an ‘N’ value?												
Yes	120 (100%)	118 (99.2%)	> 0.99	40 (100%)	39 (97.5%)	> 0.99	40 (100%)	40 (100%)	> 0.99	40 (100%)	39 (100%)	0.91
No	0 (0%)	1 (0.8%)		0 (0%)	1 (2.5%)		0 (0%)	0 (0%)		0 (0%)	0 (0%)	
Did the MRI report meet Mercury Study Group criteria?												
Yes	54 (45%)	93 (77.5%)	< 0.001‡	5 (12.5%)	27 (67.5%)	< 0.001‡	33 (82.5%)	38 (95%)	0.16	16 (40%)	28 (70%)	0.013*
No	66 (55%)	27 (22.5%)		35 (87.5%)	13 (32.5%)		7 (17.5%)	2 (5%)		24 (60%)	12 (30%)	

*ACOR* clinical audit for radiation oncology, *ICS* Institut Català de la Salut, *ICO* Institut Català d’Oncologia, *MRI* magnetic resonance imaging, *NR* not reported

*p-value 0.05 to 0.01; †*p*-value ≤0.01; ‡*p*-value ≤0.001

**Centre A: 13 cases had the distance to the anorectal union (not to the anal verge)

The outcomes for the treatment phase are detailed in Table 4. The pattern of neoadjuvant treatment is similar between centres and in both cohorts. In ACOR-2, 62.7% of the patients received a total of 50.4 Gy (LCRT), a significant difference with respect to ACOR-1 that mainly reflects an improvement in centre A.

**Table 4:** Action plan results: outcomes in treatment phase I (neoadjuvant treatment)

	Total patients			Centre A			Centre B			Centre C		
	*ACOR-1* *(N = 120)*	*ACOR-2* *(N = 120)*	*p-value*	*ACOR-1* *(N = 40)*	*ACOR-2* *(N = 40)*	*p-value*	*ACOR-1* *(N = 120)*	*ACOR-2* *(N = 120)*	*p-value*	*ACOR-1* *(N = 40)*	*ACOR-2* *(N = 40)*	*p-value*
Planned treatment												
RT + chemo	115 (95.8%)	107 (89.2%)	0.086	37 (92.5%)	34 (85%)	0.48	39 (97.5%)	39 (97.5%)	> 0.99	39 (97.5%)	34 (85%)	0.11
RT	5 (4.2%)	13 (10.8%)		3 (7.5%)	6 (15%)		1 (2.5%)	1 (2.5%)		1 (2.5%)	6 (15%)	
Type of radiotherapy												
LCRT	108 (90%)	102 (85%)	0.33	36 (90%)	32 (80%)	0.35	40 (100%)	38 (95%)	0.47	32 (80%)	32 (80%)	> 0.99
SCRT	12 (10%)	18 (15%)		4 (10%)	8 (20%)		0 (0%)	2 (5%)		8 (20%)	8 (20%)	
Type of indicated technology												
3D	117 (97.5%)	95 (79.2%)	< 0.001‡	40 (100%)	23 (57.5%)	< 0.001‡	40 (100%)	38 (95%)	0.47	37 (92.5%)	34 (85%)	0.48
VMAT	3 (2.5%)	25 (20.8%)		0 (0%)	17 (42.5%)		0 (0%)	2 (5%)		3 (7.5%)	6 (15%)	
Total dosage administered												
25 Gy	12 (10.3%)	18 (15.3%)	0.034*	4 (10.3%)	8 (21.1%)	< 0.001‡	0 (0%)	2 (5%)	0.36	8 (21.1%)	8 (20%)	0.57
45 Gy	34 (29.1%)	15 (12.7%)		34 (87.2%)	14 (36.8%)		0 (0%)	0 (0%)		0 (0%)	1 (2.5%)	
50 Gy	3 (2.6%)	6 (5.1%)		0 (0%)	0 (0%)		0 (0%)	0 (0%)		3 (7.9%)	6 (15%)	
50.4 Gy	63 (53.8%)	74 (62.7%)		1 (2.6%)	16 (42.1%)		35 (87.5%)	33 (82.5%)		27 (71.1%)	25 (62.5%)	
54 Gy	5 (4.3%)	5 (4.2%)		0 (0%)	0 (0%)		5 (12.5%)	5 (12.5%)		0 (0%)	0 (0%)	
Prolongation of treatment time (if SCRT)												
< 3 days	12 (100%)	17 (94.4%)	> 0.99	4 (100%)	8 (100%)	0.25	0 (NaN%)	2 (100%)	0.16	8 (100%)	7 (87.5%)	> 0.99
3–5 days	0 (0%)	1 (5.6%)		0 (0%)	0 (0%)		0 (NaN%)	0 (0%)		0 (0%)	1 (12.5%)	
Prolongation of treatment time (if LCRT)												
< 3 days	62 (57.4%)	58 (56.9%)	0.96	19 (52.8%)	20 (62.5%)	0.22	31 (77.5%)	27 (71.1%)	0.53	12 (37.5%)	11 (34.4%)	0.90
3–5 days	32 (29.6%)	31 (30.4%)		11 (30.6%)	10 (31.2%)		9 (22.5%)	9 (23.7%)		12 (37.5%)	12 (37.5%)	
6–7 days	4 (3.7%)	5 (4.9%)		0 (0%)	1 (3.1%)		0 (0%)	1 (2.6%)		4 (12.5%)	3 (9.4%)	
> 7 days	10 (9.3%)	8 (7.8%)		6 (16.7%)	1 (3.1%)		0 (0%)	1 (2.6%)		4 (12.5%)	6 (18.8%)	
Week day when treatment was finalised												
Monday	17 (14.2%)	7 (5.8%)	0.24	6 (15%)	1 (2.5%)	0.16	6 (15%)	3 (7.5%)	0.68	5 (12.5%)	3 (7.5%)	0.28
Tuesday	24 (20%)	22 (18.3%)		11 (27.5%)	11 (27.5%)		5 (12.5%)	6 (15%)		8 (20%)	5 (12.5%)	
Wednesday	13 (10.8%)	12 (10%)		5 (12.5%)	5 (12.5%)		2 (5%)	1 (2.5%)		6 (15%)	6 (15%)	
Thursday	24 (20%)	27 (22.5%)		8 (20%)	5 (12.5%)		11 (27.5%)	9 (22.5%)		5 (12.5%)	13 (32.5%)	
Friday	42 (35%)	52 (43.3%)		10 (25%)	18 (45%)		16 (40%)	21 (52.5%)		16 (40%)	13 (32.5%)	

*ACOR* clinical audit for radiation oncology, *LCRT* long-course radiotherapy, *SCRT* short-course radiotherapy, *RT* radiotherapy, *VMAT* volumetric modulated arc therapy

*p-value 0.05 to 0.01; †*p*-value ≤0.01; ‡*p*-value ≤0.001

A protocol for managing treatment interruptions was approved in June 2017. Compliance was 9.7%; we did not observe a significant decrease in patients with prolonged OTT over seven days (in the case of LCRT) or in the percentage of patients who received their last dose on a Monday (radiotherapy units are not active on weekends). A protocol for image-guided radiotherapy was approved in October 2016; the compliance rate was 95.8%.

There was a significant increase in the performance of the MRI restaging procedure in all centres, from 32.5 to 61.3% (Table 5). The proportion of patients in whom surgery was indicated dropped slightly, from 96.7 to 90%, reflecting an increase in patients following a conservative treatment strategy. There was also a significant improvement in the availability of the ypCRM on the anatomical pathology report (ACOR-2: 94.4%).

**Table 5:** Action plan results: outcomes in treatment phase II (restaging and surgical treatment)

	Total patients			Centre A			Centre B			Centre C		
	*ACOR-1* *(N = 120)*	*ACOR-2* *(N = 120)*	*p-value*	*ACOR-1* *(N = 40)*	*ACOR-2* *(N = 40)*	*p-value*	*ACOR-1* *(N = 120)*	*ACOR-2* *(N = 120)*	*p-value*	*ACOR-1* *(N = 40)*	*ACOR-2* *(N = 40)*	*p-value*
Was the restaging report available?												
Yes	39 (32.5%)	73 (61.3%)	< 0.001‡	0 (0%)	16 (40%)	< 0.001‡	31 (77.5%)	37 (94.9%)	0.057	8 (20%)	20 (50%)	0.01†
No	81 (67.5%)	46 (38.7%)		40 (100%)	24 (60%)		9 (22.5%)	2 (5.1%)		32 (80%)	20 (50%)	
Did the patient undergo surgery?												
Yes	116 (96.7%)	108 (90%)	0.035*	40 (100%)	33 (82.5%)	0.018*	37 (92.5%)	37 (92.5%)	> 0.99	39 (97.5%)	38 (95%)	0.22
No	3 (2.5%)	12 (10%)		0 (0%)	7 (17.5%)		3 (7.5%)	3 (7.5%)		0 (0%)	2 (5%)	
NR	1 (0.8%)	0 (0%)		0 (0%)	0 (0%)		0 (0%)	0 (0%)		1 (2.5%)	0 (0%)	
Staging (in patients undergoing surgical treatment)												
0	19 (16.4%)	20 (18.5%)	0.54	7 (17.5%)	5 (15.2%)	0.52	7 (18.9%)	9 (24.3%)	0.93	5 (12.8%)	6 (15.8%)	0.53
I	4 (3.4%)	3 (2.8%)		2 (5%)	1 (3%)		1 (2.7%)	1 (2.7%)		1 (2.6%)	1 (2.6%)	
II	62 (53.4%)	47 (43.5%)		20 (50%)	12 (36.4%)		18 (48.6%)	19 (51.4%)		24 (61.5%)	16 (42.1%)	
III	25 (21.6%)	28 (25.9%)		10 (25%)	12 (36.4%)		9 (24.3%)	6 (16.2%)		6 (15.4%)	10 (26.3%)	
IV	6 (5.2%)	10 (9.3%)		1 (2.5%)	3 (9.1%)		2 (5.4%)	2 (5.4%)		3 (7.7%)	5 (13.2%)	
ypCRM (in patients undergoing surgical treatment)												
Positive	5 (4.3%)	16 (14.8%)	< 0.001‡	1 (2.5%)	4 (12.1%)	< 0.001‡	3 (8.1%)	3 (8.1%)	0.037*	1 (2.6%)	9 (23.7%)	0.006†
Negative	71 (61.2%)	86 (79.6%)		15 (37.5%)	26 (78.8%)		28 (75.7%)	34 (91.9%)		28 (71.8%)	26 (68.4%)	
NR	40 (34.5%)	6 (5.6%)		24 (60%)	3 (9.1%)		6 (16.2%)	0 (0%)		10 (25.6%)	3 (7.9%)	

*ACOR* clinical audit for radiation oncology, *NR* not reported

*p-value 0.05 to 0.01; †*p*-value ≤0.01; ‡*p*-value ≤0.001

For the follow-up phase, adverse effects of treatment were reported on 99.8% of the visits, and these were graded for severity according to the CTCAE in 36.2% of the cases.

## Discussion

Clinical audits are a well-known tool for identifying areas to improve in the care process. This study reports the results of an action plan for improving quality in neoadjuvant treatment for locally advanced rectal cancer in three centres, based on the findings of an initial quality audit. The ACOR-1 audit revealed variability in clinical practice between centres and showed suboptimal outcomes for several standard quality indicators. ACOR-2 showed the evolution of these indicators following the implementation of quality improvement measures.

In the diagnostic phase, the MRI is the best tool to identify the patients with locally advanced tumours who would most benefit from neoadjuvant treatment [14]. All the cases audited in ACOR-2 included this test, and there was a significant improvement in the quality of the report. Specifically, there was an increase in adherence to the Mercury Group’s recommendations on reporting rectal cancer MRIs [15], including the distance from the tumour to the mesorectal fascia (CRM), reported in 61.7% of the cases in ACOR-1 and 82.5% in ACOR-2, and the distance to the anal verge or the anorectal union, reported in 68.3 and 96.7% of the cases, respectively (*p* <  0.001). Overall, the centres did not achieve the optimal level of reporting (85%), but 77.5% of the reports in ACOR-2 did meet the established criteria, compared to just 45% in ACOR-1 – a significant change. We consider that having a proforma, which would include the state of the resection margin (CRM) and the distance from the tumour to the anal verge, would be a decisive factor in achieving optimal reporting, as described by Taylor and colleagues [18, 19].

The number of patients coming to the ICO-ICS unit with a diagnosis in hand increased significantly, probably due to the centralisation policies implemented for this pathology in the region [20]. All the cases diagnosed in the ICO unit were reviewed by a tumour board to approve the therapeutic strategy.

In ACOR-2, the treatment plan adopted most frequently was radiochemotherapy, with LCRT and a dose of 50.4 Gy. Compared with the outcomes observed in ACOR-1, ACOR-2 showed greater standardisation between centres and better adherence to the approved guidelines, although some variability persists, which is consistent with other studies [21]. For example, two of the centres used SCRT in 20% of the patients, while the other did so in just 5%. This difference is probably due to the divergent approaches in patients of advanced age. One centre adheres to the strategy for avoiding surgery in frail patients, so its adoption of LCRT is more assiduous. In contrast, the other two centres favour the avoidance of chemotherapy [22, 23].

We also noted an increase in the use of volumetric modulated arc therapy (VMAT), from 2.5 to 20.8% (*p* <  0.001), and its dissemination in all three centres. Although the radiated intestinal volume may be susceptible to late toxicity, VMAT reduces acute toxicity and consequently, treatment interruptions [16]. Thus, consensus-based indications were developed for patients with tumours that infiltrated into adjacent organs, and in general when treatment of the external iliac lymph nodes and/or the groin nodes is unavoidable.

One of the action plan’s target areas was OTT. Even though special efforts were made to improve this indicator, both audits showed that the overall RT treatment time was still more than seven days longer than planned for LCRT, usually because of weekday public holidays and technical inspections of the accelerators. There were no significant changes between study periods. We also observed a non-significant improvement in the percentage of patients whose last RT session was on a Monday. To improve this aspect, a protocol for managing treatment interruptions was designed and finally approved in June 2017. The delay in approval is probably the reason that we identified only seven patients in whom it was appropriately applied (four of them in centre B). Indeed, the included patients initiated treatment only from June to December 2017. Thus, the centres still have work to do in improving adherence to the treatment. As with any measure that affects the daily work routine and entails organisational changes, achieving success in this endeavour will be challenging [24].

Another target area was the standardisation of IGRT during treatment. This measure, too, was subject to a new protocol (approved in October 2016), and adherence was 95.8%.

Once the neoadjuvant treatment is finalised, restaging (primarily based on MRI) is necessary to define the surgical technique. We observed a significant rise in the adoption of this approach in all three centres, with 32.5% of all cases undergoing MRI restaging in ACOR-1 and 61.3% in ACOR-2 (*p* <  0.001). Moreover, there is already a consolidated scientific basis permitting selected patients to follow a conservative strategy and avoid surgery, that is, ‘watch and wait’ [25, 26]. In our case, in ACOR-2, surgery was indicated in 90% of the patients, compared to 96.7% in ACOR-1 (*p* = 0.035). In 3/12 patients (25%) in whom surgery was not indicated, the reason was having cT_0_N_0_ after completing chemoradiotherapy_._

Without a doubt, the most important quality indicator in neoadjuvant treatment of rectal cancer for local control and survival is the state of the circumferential resection margin (ypCRM). This detail was not described in the anatomical pathology report for 34.5% of the surgical specimens in ACOR-1, compared to 5.6% in ACOR-2 (*p* <  0.001). Overall, 14.8% of the surgical specimens showed CRM involvement (ypCRM+), which is consistent with the literature [27, 28]. Considering the RT strategy, 13% of the LCRT treatments had ypCRM+, compared to 25% of those with SCRT treatment. We considered the proportion of 10% to be optimal.

With regard to the low-quality reporting of adverse events over the clinical course (absence of grading and aspects related to sexual function or bowel control), the specific list of CTCAE (v 4.0) criteria [15] helped to moderately improve reporting. Nevertheless, the results obtained in ACOR-2 highlight the need to double down on efforts to improve this aspect.

The ultimate aim of treatment is not only to cure disease, but also to restore the person’s dignity and achieve their social reinsertion. To this end, it is essential to understand the impact of dysfunctions and symptoms derived from treatment on patients’ quality of life [29]. Future studies are necessary to explore this aspect in the long term, applying specific validated instruments like the EORTC questionnaires for quality of life in cancer patients and for colorectal quality of life in patients with rectal cancer receiving neoadjuvant treatment.

This study has several limitations. Some are related to the design of the audit, which included only 40 clinical histories per centre, limiting the power of the assessment, especially for clinical outcomes. In addition, the period selected (especially for the ACOR-2) allowed little time for the consolidation of changes derived from the action plan. Moreover, the data collected were from clinical histories; any measure taken but not recorded on the patient’s electronic medical record would, for the purposes of the clinical audit, be non-existent.

In conclusion, this study analyses the influence of feedback from clinical audit data in improving neoadjuvant treatment procedures in rectal cancer. Our results show progress as well as areas in which additional efforts should be made, including: decreasing the percentage of positive ypCRMs; limiting treatment interruptions in order to reduce overall treatment time; and better selection of therapeutic options in elderly patients, according to frailty and mobility. In short, the audit undertaken shows total or partial correction in most areas targeted for quality improvements following the first audit, a decrease in variability between centres, and an improvement in adherence to clinical practice guidelines for rectal cancer.

## Data Availability

The datasets during and/or analysed during the current study available from the corresponding author on reasonable request.

## Abbreviations

ACOR-1: first clinical audit for radiation oncology
ACOR-2: second clinical audit for radiation oncology
CTCAE: Common Terminology Criteria for Adverse Events
EORTC: European Organisation for Research and Treatment of Cancer
ICO: Institut Català d’Oncologia
ICS: Institut Català de la Salut
IGRT: image-guided radiotherapy
IMRT: intensity-modulated radiotherapy
LART: locally advanced rectal tumour
LCRT: long course radiotherapy
MRI: magnetic resonance imaging
OTT: overall treatment time
RT: radiotherapy
SCRT: short course radiotherapy
VMAT: volumetric modulated arc therapy
ypCRM: circumferential resection margin (following neoadjuvant treatment)
